# Muscle electromyographic activity normalized to maximal muscle activity, not to M_max_, better represents voluntary activation

**DOI:** 10.1371/journal.pone.0277947

**Published:** 2022-11-21

**Authors:** Joanna Diong, Kenzo C. Kishimoto, Jane E. Butler, Martin E. Héroux

**Affiliations:** 1 School of Medical Sciences, Faculty of Medicine and Health, The University of Sydney, Camperdown, New South Wales, Australia; 2 Neuroscience Research Australia (NeuRA), Sydney, New South Wales, Australia; 3 School of Health Sciences, Faculty of Medicine and Health, The University of Sydney, Camperdown, New South Wales, Australia; 4 School of Medical Sciences, University of New South Wales, Kensington, New South Wales, Australia; Universita degli Studi di Milano, ITALY

## Abstract

In human applied physiology studies, the amplitude of recorded muscle electromyographic activity (EMG) is often normalized to maximal EMG recorded during a maximal voluntary contraction. When maximal contractions cannot be reliably obtained (e.g. in people with muscle paralysis, anterior cruciate ligament injury, or arthritis), EMG is sometimes normalized to the maximal compound muscle action potiential evoked by stimulation, the M_max_. However, it is not known how these two methods of normalization affect the conclusions and comparability of studies. To address this limitation, we investigated the relationship between voluntary muscle activation and EMG normalized either to maximal EMG or to M_max_. Twenty-five able-bodied adults performed voluntary isometric ankle plantarflexion contractions to a range of percentages of maximal voluntary torque. Ankle torque, plantarflexor muscle EMG, and voluntary muscle activation measured by twitch interpolation were recorded. EMG recorded at each contraction intensity was normalized to maximal EMG or to M_max_ for each plantarflexor muscle, and the relationship between the two normalization approaches quantified. A slope >1 indicated EMG amplitude normalized to maximal EMG (vertical axis) was greater than EMG normalized to M_max_ (horizontal axis). Mean estimates of the slopes were large and had moderate precision: soleus 8.7 (95% CI 6.9 to 11.0), medial gastrocnemius 13.4 (10.5 to 17.0), lateral gastrocnemius 11.4 (9.4 to 14.0). This indicates EMG normalized to M_max_ is approximately eleven times smaller than EMG normalized to maximal EMG. Normalization to maximal EMG gave closer approximations to the level of voluntary muscle activation assessed by twitch interpolation.

## Introduction

To compare the amount of muscle activity between people or across experimental conditions, electromyographic activity (EMG) is often expressed as a percentage of a reference value. That is, EMG is normalized. A common reference value is the maximal EMG produced during a maximal voluntary contraction. Maximal EMG can be measured consistently in most superficial limb muscles [1] and, in healthy able-bodied people, reflects maximal or near maximal neural drive [2, 3]. Thus, maximal EMG is considered to be a reproducible and meaningful reference value for EMG normalization.

Some people, however, are unable to maximally activate their muscles due to muscle paralysis from central nervous system damage [4–6], arthrogenic muscle inhibition from anterior cruciate ligament (ACL) injury [7], or arthritis [8]. This causes the EMG recorded during a maximal voluntary contraction to be submaximal. If this activity is used as the reference value for EMG normalization, the amplitude of normalized muscle activity will be *overestimated*. Precisely estimating the amplitude (magnitude) of normalized muscle activity is important, as small amounts of involuntary muscle activity common in neurological conditions can substantially impair physical function [9–11], and the amplitude of muscle activity contributes to motor control in gait after ACL reconstruction [12, 13]. In fact, laboratory studies on passive joint movement often include data recorded in the presence of EMG using nominal cut-offs (anywhere from 1 to 10% of normalized EMG) [14–17]. This means that passive mechanical properties of muscles, at times, were investigated under active conditions. Low-grade involuntary muscle activity was more likely to bias these findings if data recorded in the presence of higher amplitudes of EMG were included.

One solution, which we recently proposed for the human plantarflexor muscles [18], is to estimate maximal muscle activity using submaximal muscle activity and voluntary muscle activation. Measurement of EMG across the full range of voluntary muscle activation allowed us to derive predictive equations to describe the log-linear relationship between these two variables. Although promising, this approach has yet to be validated and is experimentally complicated as it requires accurate measures of twitch force.

Another solution is to use a maximal compound muscle action potential, better known as a maximal M wave or M_max_, as the reference value for EMG normalization. This evoked response, produced by maximal or supramaximal electrical stimulation of a motor nerve, is not affected by impaired voluntary muscle activation [9, 19, 20] and accounts for changes in muscle size or denervation. Since it reflects the synchronized activity of all motor units from a muscle [21], M_max_ is much larger in amplitude than the EMG produced during a maximal voluntary contraction. Thus, M_max_ is a largely reproducible reference value for EMG normalization, but is it a meaningful one?

M_max_ is well suited as a reference value for other evoked responses such as H-reflexes and motor evoked responses elicited by transcranial magnetic stimulation [22, 23]. However, it is less clear how M_max_ is related to naturally occurring muscle activity—whether voluntary or involuntary—and how it relates to voluntary muscle activation. This is important when trying to determine the normalized muscle activity measure that best reflects the natural force-generating capacity of a muscle. For example, what does it mean to record EMG with an amplitude of 5% of M_max_? Is this comparable to EMG with an amplitude of 5% of maximal EMG? How is EMG normalized to M_max_ related to the level of voluntary muscle activation assessed by twitch interpolation? The answers to these questions are important as they influence the interpretation of findings from studies in people with neurological conditions where normalization to M_max_ is used [9, 19, 20], and studies in sports medicine that normalize voluntary background [24] and maximal EMG [25–30] to M_max_.

Our recent findings indicate that triceps surae muscle EMG normalized to maximal EMG in healthy able-bodied people has a log-linear relationship with joint torque and voluntary muscle activation assessed by twitch interpolation [18]. That is, EMG amplitude *does not* scale linearly with either the strength of a muscle contraction or, as is often suggested [31], the proportion of a muscle that is active. The log-linear nature of this relationship means that for triceps surae muscles, changes in (raw or normalized) EMG amplitudes that are not log-transformed are not proportional to changes in force generation or voluntary muscle activation. Moreover, it is not clear whether EMG normalized to maximal EMG or to M_max_ is comparable, and which of the two better represents the level of voluntary muscle activation.

To address this fundamental question, we re-analysed plantarflexor muscle activity and voluntary muscle activation from our recently published study [18]. Specifically, we systematically investigated the relationship between voluntary muscle activation and EMG across voluntary contraction intensities that ranged between 1 and 100% maximal torque, which were normalized in two different ways, (i) to maximal EMG and (ii) to M_max_, to provide insight into the appropriateness of using M_max_ in EMG normalization.

## Materials and methods

Methods have been described in full elsewhere [18]. Briefly, data were collected from 25 participants [mean (SD) unless otherwise stated: age 29 (10) years; 18 males, 7 females; height 1.74 (0.08) m; weight 73.6 (13.5) kg] who could achieve ≥80% voluntary muscle activation assessed with twitch interpolation when performing attempted maximal plantarflexion contractions. The procedures conformed to the Declaration of Helsinki and were approved by The University of Sydney Human Research Ethics Committee (2018/1007). Informed consent was obtained in writing from all participants. In the interests of research reproducibility and transparency, the protocol was registered on the Open Science Framework, and de-identified data and computer code used for analysis are available from the public repository.

### Experimental set-up and protocol

Participants sat with the right knee flexed 90° and the ankle in 5° of dorsiflexion to reduce slack in the plantarflexor tendon. The knee, ankle and foot were firmly stabilized on a custom footplate [3]. The axis of rotation of the footplate was aligned with the lateral malleolus, and torque about this axis was measured as the product of plantarflexor force (MPL-50, Transducer Techniques, Temecula, CA) and the moment arm of the footplate, discounting the weight torque of the footplate.

Pairs of surface electrodes (diameter: 10 mm, spacing: 30 mm) were used to record EMG from the three plantarflexor muscles: soleus, medial and lateral gastrocnemius muscles. A ground electrode was placed over the lower third of the anterior surface of the tibia.

Supramaximal stimuli were delivered to the tibial nerve (single pulses, 200 μs pulse width; Digitimer, DS7AH, Welwyn City Gardens, UK) via a ball cathode surface electrode (diameter: 23 mm) secured firmly in the middle of the popliteal fossa, and a surface anode electrode (diameter: 10 mm) placed over the patella. The current intensity used was 120% of the current required to maximally activate the plantarflexor muscles; this was determined online from the peak-to-peak amplitude of the M waves from soleus muscle and the amplitude of the evoked twitch.

Participants performed maximal voluntary isometric contractions of the plantarflexor muscles to determine the maximal voluntary plantarflexion torque. The peak torque across five maximum voluntary contractions (MVCs) was used as the best estimate of maximal voluntary plantarflexion torque. This torque was used to set the submaximal target torques used in each trial.

Twitch interpolation was then used to assess each participant’s ability to activate the plantarflexor muscles maximally during MVC [32].

Ankle plantarflexion torque and plantarflexor muscle EMG were recorded as participants performed a single isometric voluntary ankle plantarflexion contraction for 3 s at 1, 5, 10, 15, 25, 50, 75, 90, 95, 100% of MVC, in random order. Single pulse stimulation was delivered to the tibial nerve to evoke a superimposed twitch during these isometric contractions, and at ∼2 s after these contractions (i.e. at rest). Voluntary muscle activation was calculated from the increase in torque evoked by the superimposed twitch during the peak torque relative to the increase in torque evoked by the twitch at rest:

$$1-(\frac{superimposed\;twitch}{resting\;twitch})*100$$

### Data recording and analysis

Twitch amplitude was calculated as the difference between minimal and maximal torques about the single pulse stimulation. Resting and superimposed twitch amplitudes were used to calculate plantarflexor muscle activation for each trial.

EMG during submaximal and maximal isometric plantarflexion contractions and the M_max_ (or maximal M wave) at rest were measured for the soleus, medial and lateral gastrocnemius muscles. For each plantarflexor muscle, the level of EMG recorded during submaximal isometric plantarflexion contractions was calculated as the root-mean-square (RMS) of the EMG signal over 50 ms prior to the single pulse stimulation.

Maximal EMG was calculated as the RMS EMG over 50 ms prior to maximal torque across the five plantarflexion MVCs performed at the beginning of the experiment.

**Fig 1:**
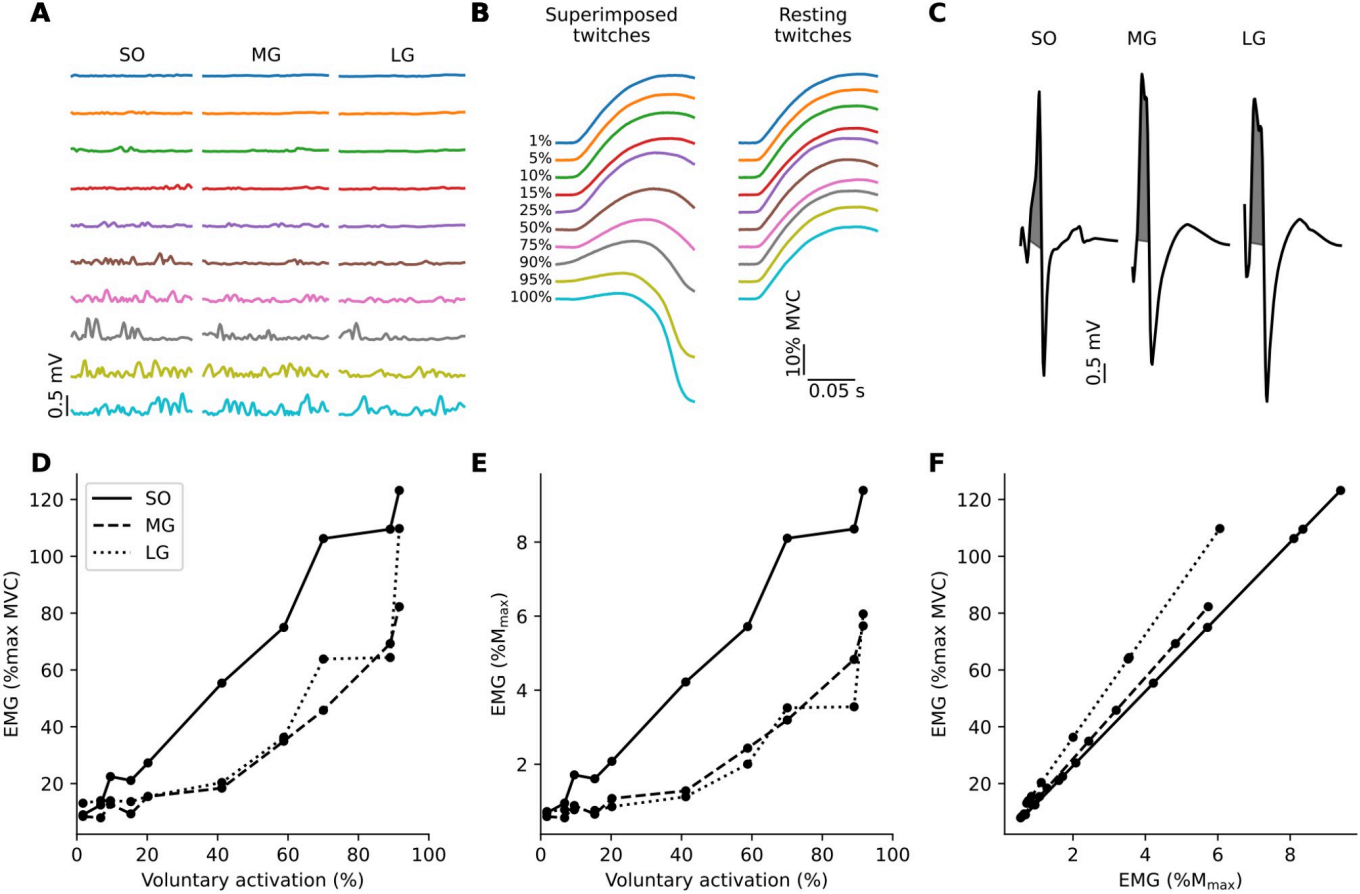
Data from an individual participant. *A*: individual traces of root-mean-square (RMS) electromyographic activity (EMG) over 50 ms prior to single pulse stimulation during contractions at each % of maximal voluntary contraction (MVC). *B*: superimposed and resting twitches evoked by supramaximal tibial nerve stimulation for all trials at each % of MVC. Twitches are offset vertically for clarity. Colors in panels *A* and *B* indicate corresponding trials. *C*: maximal M waves from soleus, medial and lateral gastrocnemius. The RMS EMG was calculated from the first phase of the maximal M wave (gray shaded regions) using the method by Thomas [20]. The lower bounds of the gray shaded regions that indicate the first phase of the M waves are not horizontal because when the signal crossed 0, the closest sampled time points are sometimes slightly above or below 0 V. *D*: EMG normalized to maximal EMG as a function of voluntary activation. *E*: EMG normalized to M_max_ as a function of voluntary activation. *F*: EMG normalized to maximal EMG as a function of EMG normalized to M_max_. The slope of each line indicates the scaling factor for that muscle. Legend for line styles in panel *D* indicates corresponding muscles in panels *D*, *E* and *F*. Data from individual trials in these panels are shown (black circles).

The M_max_ for each muscle was identified as the largest M wave evoked by tibial nerve stimulation during the set up for supramaximal stimulation intensity, where single pulse stimulations were delivered at increasing current intensities. This was the M wave associated with no further increase in plantarflexion torque as current intensity increased. The size of M_max_ used to normalize the EMG was calculated as the RMS EMG over the first phase of the maximal M wave using the method by Thomas [20] (Fig 1C). The first phase of the M wave signal was rectified and interpolated to identify the two time points at which the signal crossed 0 volts: from negative to positive volts, and from positive to negative volts. RMS EMG over the first phase of the maximal M wave was calculated between these time points.

EMG during submaximal isometric plantarflexion contraction was normalized separately to both the maximal EMG and the M_max_. Data were analyzed using custom-written scripts in Python v3.8.

### Statistical analysis

For each muscle, EMG normalized to maximal EMG was plotted as a function of EMG normalized to M_max_. The slopes of these lines indicate the increase in EMG activity normalized to maximal EMG for each 1% increase in EMG normalized to M_max_. That is, the magnitudes of the slopes indicate the extent to which the level of EMG amplitude is overestimated by normalization to M_max_ compared to maximal EMG. EMG normalized to either maximal EMG or M_max_ were also plotted as a function of voluntary activation assessed by twitch interpolation to determine which normalization method better reflected voluntary activation.

Histograms of slopes from individual participants showed data were not normally distributed. Thus, slope values were transformed using the natural log. The mean, 95% confidence interval (CI) and 95% prediction interval of the slopes in log-units were determined, and the values were back-transformed to natural units. The prediction interval was determined using t = 2.064 standard deviations from the mean (with n–1 = 24 degrees-of-freedom). The prediction interval shows the range in which a future participant’s slope will fall, whereas the confidence interval shows the precision about the mean slope. The width of the 95% prediction interval indicates uncertainty about the mean slope as well as variability in slopes between participants; it is always wider than the 95% CI [33].

## Results

The mean maximal voluntary plantarflexion torque was 148 (SD 41) Nm (range 74 to 230 Nm), and the mean voluntary muscle activation was 90 (SD 5)% (range 81 to 98%). Signals from a single participant at all stages of analysis are shown (Fig 1).

**Fig 2:**
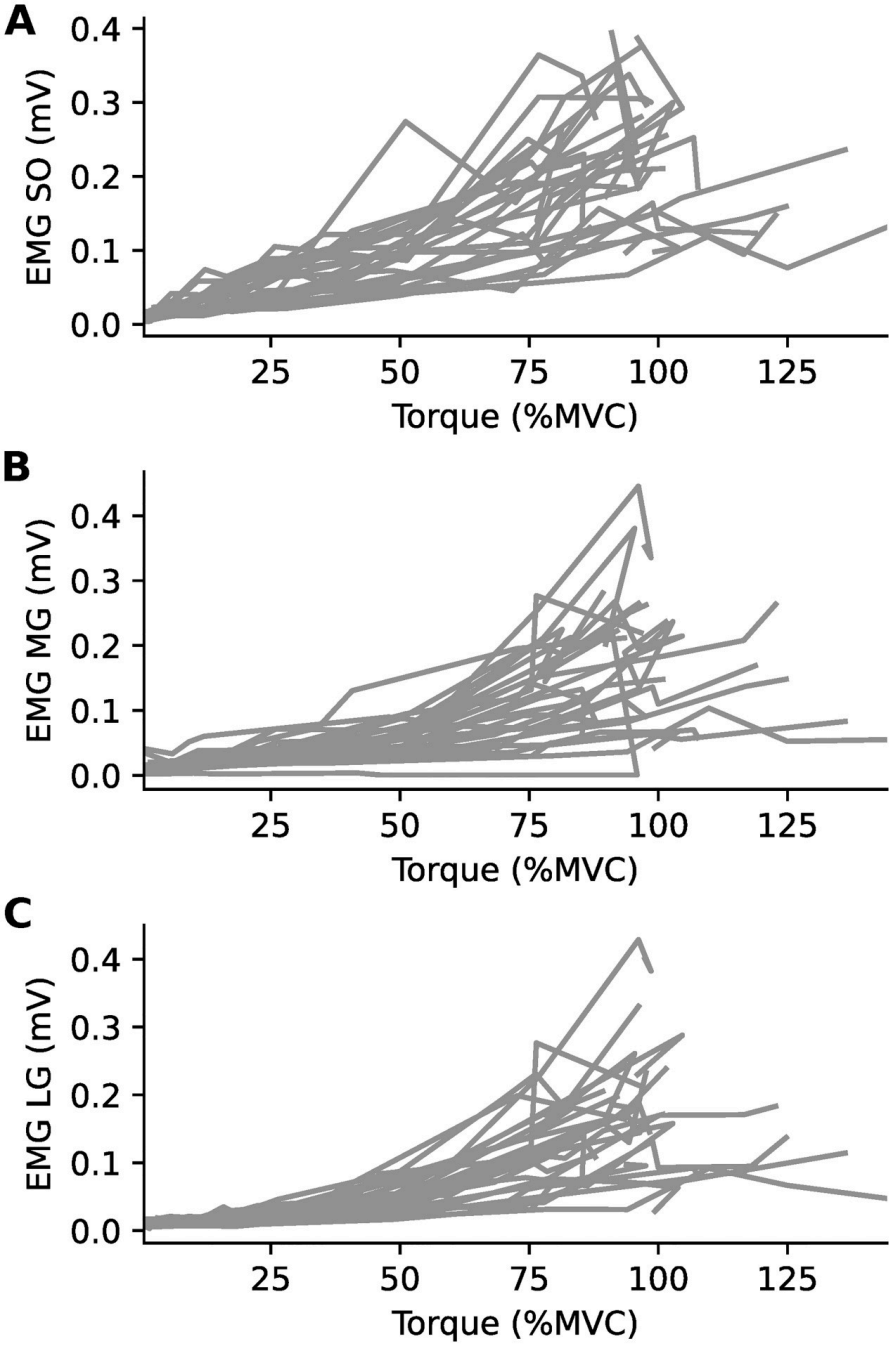
Non-linear relationship between EMG and torque. Electromyographic activity (EMG) in mV and ankle torque data from all participants (n = 25, 18 males) for *A*: soleus (SO), *B*: medial gastrocnemius (MG) and *C*: lateral gastrocnemius (LG) muscles. Torque is normalized to the maximal torque in the maximal voluntary contractions (MVCs) performed at the start of the protocol.

**Fig 3:**
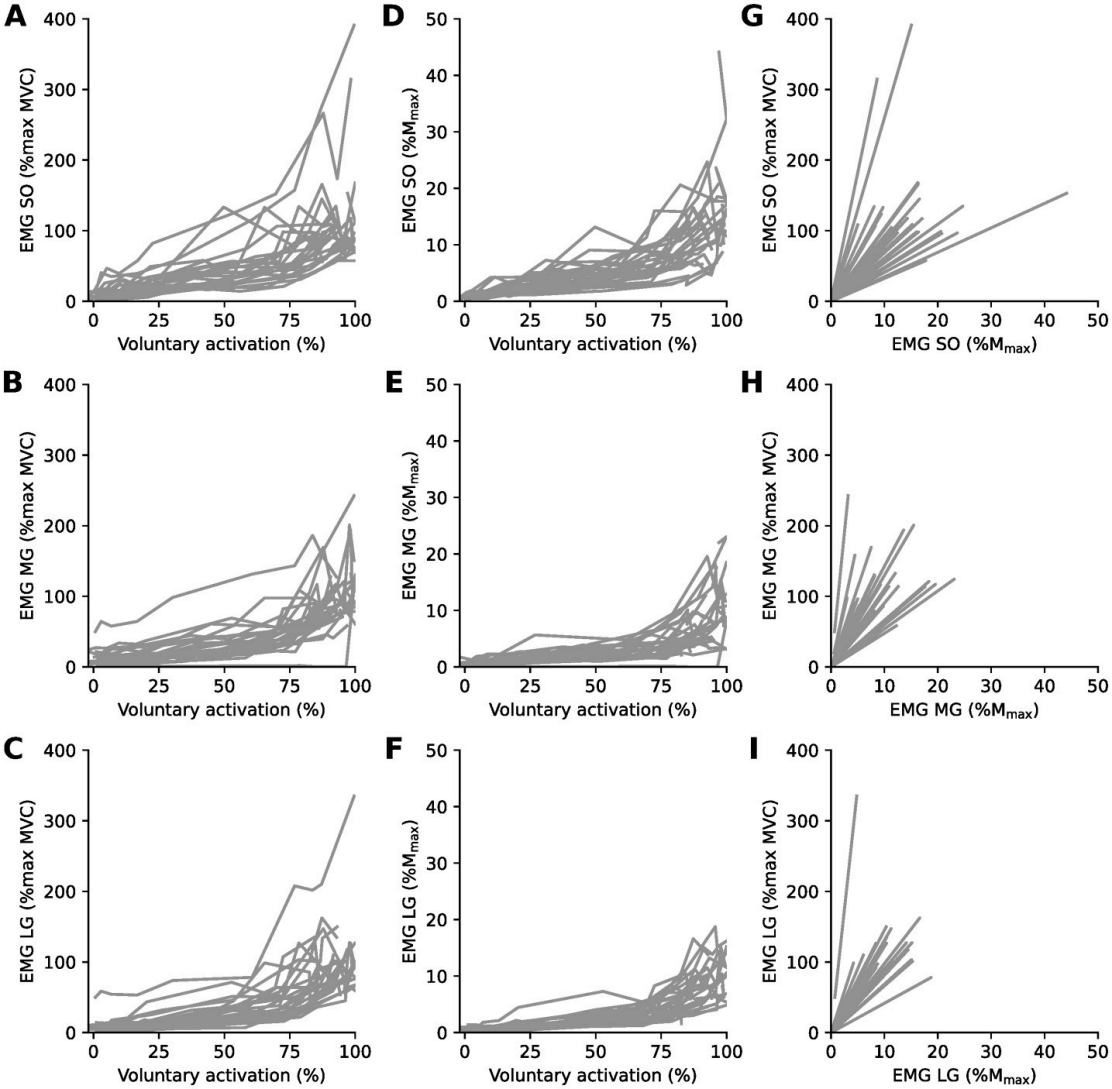
Effect of normalization method on the estimated level of muscle activation. Data from each participant (n = 25, 18 males) of electromyographic activity (EMG) in each contraction level normalized to maximal EMG as a function of voluntary activation (panels *A-C*), EMG normalized to M_max_ as a function of voluntary activation (panels *D-F*), and EMG normalized to maximal EMG as a function of EMG normalized to M_max_ (panels *G-I*) for soleus (SO, panels *A, D, G*), medial gastrocnemius (MG, panels *B, E, H*) and lateral gastrocnemius (LG, panels *C, F, I*) muscles. Slopes of lines in panels *G-I* indicate scaling factors for participants and muscles.

For all three plantarflexor muscles, the EMG recorded during submaximal contractions increased as torque increased (Fig 2), although this relationship became more variable across participants at higher torques. Likewise, EMG normalized to either maximal EMG (Fig 3A–3C) or M_max_ (Fig 3D–3F) increased as voluntary muscle activation increased, and this in a non-linear fashion.

Across participants, the amplitude of EMG normalized to M_max_ was approximately one eleventh of the amplitude of EMG normalized to maximal EMG. The relationship between these two forms of EMG normalization was associated with large average slope values (Fig 3G–3I, Table 1). Of note, the precision of slope estimates was moderate, and there was substantial variability between participants (Table 1).

**Table 1:** Means, 95% confidence intervals (CI) and 95% prediction intervals of slopes of electromyographic activity (EMG) normalized to maximal muscle activity as a function of EMG normalized to the M_max_ in the three plantarflexor muscles. Slopes are ratios with no units.

	Mean	95% CI	95% prediction interval
Soleus	8.7	6.9 to 11.0	2.5 to 30.3
Medial gastrocnemius	13.4	10.5 to 17.0	3.7 to 47.9
Lateral gastrocnemius	11.4	9.4 to 14.0	4.0 to 33.0

For all participants, EMG normalized to maximal EMG was the better estimate of voluntary muscle activation than EMG normalized to M_max_ (Fig 3A–3F). To illustrate this, EMG normalized to maximal EMG, EMG normalized to M_max_, and the level of voluntary activation at each intensity of muscle contraction are shown in Table 2.

**Table 2:** Means (SD) of voluntary muscle activation (VA) and the electromyographic activity (EMG) in soleus (SO), medial gastrocnemius (MG) and lateral gastrocnemius (LG) normalized to maximal EMG (%max EMG) and normalized to M_max_ (%M_max_). EMG normalized to maximal EMG better estimates voluntary muscle activation than EMG normalized to M_max_.

Torque (%max)	VA (%)	EMG (%max)			EMG (%M_max_)		
		SO	MG	LG	SO	MG	LG
1	1.1 (1.4)	6.6 (3.5)	11.3 (10.5)	8.7 (9.4)	0.7 (0.3)	0.7 (0.3)	0.6 (0.3)
5	4.5 (1.8)	12.1 (10.9)	12.0 (12.5)	9.7 (10.9)	1.1 (0.5)	0.7 (0.3)	0.7 (0.3)
10	9.5 (3.3)	15.0 (10.3)	13.8 (11.5)	10.8 (9.9)	1.5 (0.9)	0.9 (0.4)	0.8 (0.3)
15	16.4 (4.2)	18.8 (11.7)	16.4 (13.3)	12.7 (13.3)	2.0 (0.8)	1.1 (0.6)	1.0 (0.5)
25	29.0 (7.3)	28.4 (16.2)	22.1 (18.9)	17.8 (14.6)	3.3 (1.8)	1.5 (1.0)	1.4 (0.8)
50	52.8 (9.0)	47.3 (32.3)	34.0 (25.0)	28.5 (17.7)	5.1 (2.6)	2.4 (1.1)	2.4 (1.4)
75	73.7 (8.5)	61.3 (34.5)	47.4 (26.4)	49.3 (38.5)	7.0 (4.0)	3.9 (2.7)	4.1 (2.1)
90	86.4 (8.6)	103.6 (53.8)	86.8 (38.8)	84.0 (36.6)	11.8 (5.0)	7.3 (4.9)	7.6 (3.9)
95	91.8 (5.3)	101.8 (44.2)	96.2 (44.5)	97.1 (37.1)	12.7 (6.8)	8.0 (5.2)	8.9 (3.9)
100	95.5 (4.9)	116.4 (76.8)	100.7 (40.5)	99.9 (55.4)	13.6 (8.4)	8.6 (5.3)	9.0 (4.0)

VA: voluntary activation; EMG: electromyography; SO: soleus muscle; MG: medial gastrocnemius muscle; LG: lateral gastrocnemius muscle

## Discussion

Normalization of EMG to maximal EMG provides a better estimate of voluntary muscle activation in the plantarflexor muscles. Intuitively, EMG amplitude expressed as a percentage of maximal EMG during a maximal voluntary contraction is also functionally more relevant and meaningful than EMG amplitude expressed as a percentage of the synchronized activity of all motor units in a muscle. However, normalization of EMG to M_max_ is a potentially useful alternative in people with impaired voluntary activation. Another benefit of using maximal M waves to normalize EMG is that it is a relatively reliable measure, more so than maximal EMG. However, normalizing to M_max_ substantially underestimates EMG amplitude compared to normalization to maximal EMG and voluntary activation. The extent of this underestimation differed across muscles and was variable across participants.

The amplitude of EMG normalized to M_max_, when measured relative to maximal voluntary contraction, is underestimated by a factor of ∼8-13 times and thus, does not accurately quantify the true proportion of a muscle that is activated. The underestimation applies to studies on people with neurological conditions where maximal muscle activity could not be reliably obtained and normalization to M_max_ is used [9, 19, 20]. This underestimation also applies to findings from studies in sports medicine that normalize background [24] and maximal voluntary EMG [25–30] to M_max_ in healthy able-bodied people.

Normalization of muscle activity to M_max_ is likely to mask small but functionally important amounts of muscle activity. For instance, small amounts of involuntary muscle activity are common in people with neurological conditions [10, 11] and these substantially limit passive joint range of motion [34, 35]. On average, passive ankle dorsiflexion decreases by more than 2 deg for each 1% increase in involuntary muscle activity, relative to maximal muscle activity [34]. However, when normalized to M_max_, this involuntary muscle activity may be mistakenly interpreted as small and inconsequential. In studies on people with ACL injury or arthritis, EMG is often normalized to maximal voluntary contraction [36, 37]. However, the inability to maximally activate their muscles during attempted maximal voluntary contraction likely overestimates the normalized EMG amplitudes. Consequently, a suitable alternative is still needed to precisely quantify amplitudes of muscle activity in people with these conditions.

The extent to which EMG amplitude is underestimated by normalization to M_max_ differed across muscles; it was greatest in the medial gastrocnemius muscle and smallest in the soleus muscle. This suggests that strategies to correct for this underestimation must be muscle specific; it would not be appropriate to use a mean slope pooled across several muscles as a scaling factor. More problematic is the lack of precision of mean slope estimates caused by variability between people in all three muscles. Again, this uncertainty was greatest in the medial gastrocnemius muscle and smallest in the soleus muscle. Thus, scaling factors based on mean slopes, even if they are muscle specific, are likely to over- or under-correct EMG amplitude. This variability may be due to differences across people in the positioning of the recording electrodes relative to the motor points of the muscles, which can affect the size of the M_max_, potentially to a greater extent than it would affect EMG produced during voluntary or involuntary contractions.

In this study, we determined the extent to which EMG amplitude may be underestimated in people with intact voluntary muscle activation. Ideally, if there had been little between-participant variability, future studies would have been able to use our mean slope values as scaling factors to express EMG amplitude from plantarflexor muscles in physiologically and functionally relevant units (i.e. % of maximal muscle activity). However, our results indicate that such an approach would lead to imprecise results. For example, we found that in the medial gastrocnemius muscle, EMG normalized to M_max_ underestimates EMG normalized to maximal EMG by a factor of ∼13. However, we can be 95% confident that the true value of this is as small as 10 or as large as 17. This means that muscle activity with an amplitude of 1% M_max_ would, on average, correspond to 13% of maximal EMG, although the true value could be as small as 10% or as large as 17%. What is an acceptable amount of uncertainty for such estimates? There is no simple answer to this question. Investigators and clinical practitioners should assess the strength (interpretability) and weakness (uncertainty, complexity of interpretation) of this scaling approach.

In summary, the amplitudes of plantarflexor muscle activity obtained either by normalization of EMG to M_max_ or to maximal EMG are not comparable. Normalization of EMG to maximal EMG better reflects voluntary muscle activation assessed by twitch interpolation, and is more meaningful. The relationship between muscle activity and voluntary muscle activation is non-linear, regardless of how muscle activity is normalized. Normalization of EMG to M_max_ systematically underestimates muscle activity or voluntary muscle activation by a factor of ∼11. It is not possible to precisely correct for this underestimation with an average scaling factor because of variability between people. Therefore we recommend, where possible, that EMG normalized to M_max_ should not be used to estimate the level of muscle activation. EMG amplitudes from the two normalization approaches should be used separately to compare outcomes between participants or conditions, but comparisons of EMG amplitude should not be made between normalization approaches.
